# Loline Alkaloid Effects on Gastrointestinal Nematodes

**DOI:** 10.3390/ani12080996

**Published:** 2022-04-12

**Authors:** Kelly Ann Froehlich, Robin McAnulty, Andy Greer

**Affiliations:** 1 Department of Animal Science, College of Agriculture, Food and Environmental Sciences, South Dakota State University, Brookings, SD 57007, USA; 2 Department of Agricultural Sciences, Faculty of Agriculture and Life Sciences, Lincoln University, Lincoln 7647, New Zealand; robin.mcanulty@lincoln.ac.nz (R.M.); andrew.greer@lincoln.ac.nz (A.G.)

**Keywords:** alkaloid, loline, parasites, nematoda

## Abstract

**Simple Summary:**

Many plants contain natural secondary compounds that can exert medicinal effects. Parasitism in sheep is an animal welfare issue and affects performance. Much is unknown about natural plant compounds and how they could potentially serve as an alternative to traditional dewormers. Therefore, a series of preliminary studies conducted in the lab and in lambs were used to determine potential anti-parasitic effects of loline. Loline is an alkaloid found naturally in many fescue-type grasses and is considered non-toxic to mammals. Results from the studies were mixed. Despite potential promising results in the lab, there was limited evidence to support an anti-parasitic effect of loline in lambs. Discrepancies between lab and lamb studies were potentially a result of loline contact time with parasite larvae, mode of ingestion or the forms of loline present.

**Abstract:**

Loline, an alkaloid with several derivatives, has suggested antimicrobial and anthelmintic properties. Therefore, loline was investigated as a natural anthelmintic against *Trichostrongylus colubriformis*, *Teladorsagia circumcincta*, and *Haemonchus contortus*. Preliminary in vitro studies had reduced L3 *T. circumcincta* establishment but no effect on L3 *T. colubriformis* larvae migration or *H. contortus* establishment. While loline-treated lambs had lower establishment of L4 and adult *T. circumcincta* and L4 *T. colubriformis*, L4 and adult *H. contortus* appeared unaffected. Following preliminary study, an in vivo experiment examined lambs infected with a mix of L4 *T. circumcincta*, *T. colubriformis*, and adult *H. contortus*. These lambs were treated with either a loline seed extract (LOL, *n* = 7), nothing (CON, *n* = 7), or a non-loline seed extract (NIL, *n* = 2). There were no differences in worm burdens, fecal egg counts, weight gain, or feed intake between treatments. However, an average growth efficiency (kg LWG/kg DM intake) was detected (*p* = 0.01) in CON (0.18) which was less than LOL (0.24) or NIL (0.23). There was limited evidence to support an in vivo anti-parasitic effect of loline despite in vitro studies indicating potential benefits. Discrepancies between in vivo and in vitro studies results were potentially a result of loline contact time with larvae, mode of ingestion or the forms of loline present.

## 1. Introduction

Reliance on an anthelmintic to control gastrointestinal parasites in livestock may be reduced by using the pharmaceutical activities of some plants [1,2,3,4]. Plants can contain a wide array of secondary metabolites arranged in a variety of chemical classes [5,6] that may provide an alternative, cost-effective treatment to gastrointestinal parasites, limiting both drug use and development of anthelmintic resistance [1,7]. Any plant part can contain secondary metabolites produced from naturally occurring symbiotic microorganisms (bacteria, fungi, actinomycetes, or virus) called endophytes [8]. As a result, plants may be a novel source of bioactive compounds although research is needed to identify effective compounds to integrate into parasite control programs [6] without long-term detrimental effects on livestock health or well-being [9]. One suggested bioactive compound is loline, an alkaloid produced by *Epichloë* fungi in fescue grass species and is suggested to possess antimicrobial, insecticidal, and anthelmintic properties [10,11,12] while considered non-toxic to mammals [13,14]. Further research has shown minimal effect to rumen microbes [15]. Furthermore, loline appears to remain intact during digestion [15], and little loline is passively absorbed from the gastrointestinal tract of lambs [15]. Therefore, loline has the availability to have a pharmaceutical effect on parasites in the gut without negatively affecting livestock performance. However, further research is needed to determine if loline has a biological effect against parasites. There are several naturally occurring loline derivatives: N-formyl loline (NFL), N-acetyl loline (NAL), N-acetyl norloline (NANL), and N-methyl loline (NML). Specifically, NFL, the predominant alkaloid in loline-containing plants [16], is a contact and ingested broad-spectrum insecticide [17]. However, research has been focused on a mix of loline derivatives. Larvae development of a variety of insects [18,19,20,21,22,23], as well as egg hatching and larval motility of *Trichostrongylus colubriformis* and *Teladorsagia circumcincta* nematodes [12] have been affected by a mix of loline extracts. Specifically, a combined NAL and NFL is nematocidal to the plant parasitic nematode *Pratylenchus scribneri* at 100 and 250 µg/mL concentration [24]. Therefore, the objective of this study was to investigate and validate using loline as a natural anthelmintic for the gastrointestinal nematodes in sheep of *T. colubriformis*, *T. circumcincta*, and *Haemonchus contortus* through a series of in vitro and in vivo studies.

## 2. Materials and Methods

Several in vitro and in vivo studies (Table 1) were performed to determine anthelmintic effects of the alkaloid loline on gastrointestinal nematodes. Throughout the experiments, Meadow fescue seed (Barrier U2, Cropmark Seeds Ltd., Rolleston, New Zealand) with or without loline alkaloids was used as an extract. Extracts were made by grinding the seeds through a 1 mm sieve (ZM200, Retsch) at 18,000 RPM. Loline-containing seed contained 16.7 mg/g of total loline. Seed extracts were prepared as described by [15].

### 2.1. In Vitro Experiment 1: Loline Effects on Larval Migration

Anthelmintic effects on *T. colubriformis* L3 larvae migration were determined with eight treatments of either NIL or LOL seed extract at serial dilutions.

*T. colubriformis* larvae were exsheathed using 160 µL of a hypochlorite solution in a 15 mL tube. Larvae were washed three times, twice with water, and one final washing with a 0.85% NaCl to remove excess hypochlorite. For each wash, tubes were filled to 10 mL and were centrifuged at 1300× *g* and the supernatant decanted. Centrifugation occurred in short 10–15 s pulses to prevent larvae clumping. After the final NaCl washing, larvae were re-suspended using a 1 mL syringe by drawing and then forcefully expelling larvae back into the tube and adjusting so that 100 µL contained 200–250 larvae. A serial dilution of loline extract was made (16,000, 8000, 4000, and 2000 ppm) and larvae were incubated in each concentration at 37 °C for 2 h. The same amount of NIL extract was used and diluted as the loline extract to serve as control. At the end of the two-hour incubation, 1 mL of the different loline/NIL extract concentrations was pipetted into a 24-well plate in duplicate down an inner tube that contained a nylon mesh filter of 25 µm fitted into the plate wells. Larvae were again incubated at 37 °C for 2 h, after which filters were carefully removed from the plate wells. Remaining larvae inside of the nylon mesh filters were washed into a new well as the ‘retained’ larvae. Both retained and larvae that migrated through the mesh filter were stained with iodine and counted using an inverted microscope.

The experiment was replicated twice on separate days; and each time, samples were duplicated. Percent larvae migration was calculated by number of larvae migrated divided by the number of total larvae (migrated plus number larvae retained) × 100. All data were analyzed and compared using ANOVA in Genstat (18th edition). Loline effects on larvae migration were compared between extract (LOL or NIL) and concentration (16,000, 8000, 4000, and 2000 ppm) were considered the factors.

### 2.2. In Vitro Experiment 2: Establishment of T. circumcincta in Abomasal Tissues

Establishment of exsheathed *T. circumcincta* L3 larvae in abomasal tissues was determined using an in vitro direct challenge method as described by Jackson, Greer [25]. Experiment was replicated 3 times, and the treatments were either abomasal tissues of a lamb fed a loline lace milk (LOL) or loline-naïve lamb tissue (CON) incubated with *T. circumcincta*.

Abomasal tissues were sourced from two, 12-week-old suckling lambs. One lamb was milk fed loline at 52.5 mg/kg 14 h prior, the other served as a loline-naïve lamb. Abomasal tissues were removed immediately after slaughter, emptied of contents, and washed with physiological saline solution removing the majority of digesta. Three, 2 × 2 cm sections of abomasal tissue from the fundic folds were removed from each sheep and placed into a well of a 6-well plate with the mucosal surface facing up. Warm Hank’s medium was added to the wells surrounding but not submerging abomasal tissues. A barrel of a 10 mL syringe with needle end removed was placed into the center of the tissue providing an isolation cylinder to contain the *T. circumcincta* larvae. The syringe barrel was held in place by the lid of the 6-well plate secured with rubber bands. Exsheathed *T. circumcincta* L3 larvae in 0.5 mL of saline was placed into the syringe barrel chamber onto the mucosal surface of the abomasal tissues. The larvae were exsheathed as described previously and adjusted so that a 1 mL contained 4000 larvae. Once larvae were placed onto the abomasal mucosal surface plates were transferred to a container with a lid, which was gassed with pure oxygen for one minute. The container was sealed to maintain the high oxygen concentration and placed into a dark incubator maintained at 38 °C for 3 h. From time of slaughter to placement in incubator was no longer than 20 min.

Following the 3 h incubation, the syringe chamber and abomasal tissues were rinsed with physiological saline into 50 mL centrifuge tubes removing any larvae not associated with the abomasal tissue and were vigorously washed by immersing 30 times in 25 mL of saline. Tissues were placed in a separate 50 mL centrifuge tube and digested with 50 mL of 1% pepsin, 1% HCL solution at 38 °C for 12 h. All samples were adjusted to 50 mL. A 4% sample was taken and counted under a microscope, and estimated tissue-associated populations were calculated by adding together worm counts and dividing digest counts by total multiplied by 100.

All data were analyzed in Genstat (18th edition) using a two-sample t-test with tissues from LOL- or CON-fed lambs as factors and percentage establishment of larvae in abomasal tissue as the response variable.

### 2.3. In Vitro Experiment 3: Establishment of H. contortus in Abomasal Tissues

Establishment of *H. contortus* in abomasal tissues was determined using the same method as described in in vitro experiment 2. The treatments were either abomasal tissues of a lamb fed a loline lace milk or loline-naïve lamb tissue incubated with *H. contortus*. Abomasal tissues were sourced from three, 14–15-week-old suckling lambs. Two lambs were milk fed loline at 52.5 mg/kg 14 h prior, the other served as a loline-naïve lamb.

Data were analyzed in Genstat (18th edition) using a two-sample t-test with tissues from LOL- or CONfed lambs as factors and percentage establishment of larvae in abomasal tissue as the response variable.

### 2.4. In Vivo Study 1: Loline Effects against T. circumcincta and T. colubriformis

Loline seed extract was used to examine anthelmintic effects of the L4 and adult parasite stages of *T. circumcincta* and *T. colubriformis* in four sets of twin lambs (eight lambs total) that were approximately six weeks old (Lincoln University Animal Ethics Committee #2018-34). Lambs were still suckling and housed in four individual pens for each set of twins with their dam. Ewes and lambs were fed ad libitum lucerne pellets and had free access to water. Lambs were treated with an anthelmintic at 2 mL per 15–20 kg (Zolvix Plus, PGG Wrightson, Christchurch, New Zealand) and 10 days post treatment all lambs were infected with 10,000 L3 *T. colubriformis* and concurrently four lambs chosen at random were also dosed with 5000 L3 *T. circumcincta*. There were two treatments, loline-treated lambs and non-loline-treated lambs.

The four loline treatment lambs were trained to drink milk from a bottle with a teat to stimulate the esophageal groove reflex delivering milk directly to the abomasum and bypassing the rumen. Two bottle lambs were infected with *T. circumcincta* and *T. colubriformis* and two were infected with only *T.colubriformis*. At 10 days post dosing parasites, two lambs (only one was infected with *T. circumcincta*) were offered 200 mL milk twice, 60 h apart containing loline in a seed extract delivering 52.5 mg/kg LW [14]. This was to determine the effect of loline against L4 larvae. Loline dose was based on calculations from Gooneratne, Patchett [14], an estimate representing the daily loline consumption of a sheep grazing meadow fescue grass. The timing between dosing was chosen due to loline metabolites in sheep urine plateauing at 60 h post dosing [14]. The remaining two lambs were offered 200 mL milk twice 60 h apart with the same loline rate at 23 days post larvae infection examining the effect of loline on adult parasites. 

Fecal samples were taken directly from the rectum at the start and the end of the experiment and analyzed for concentration of nematode eggs. Feces (1.7 g) were added to a jar with 5 mL water and allowed to soften overnight. Following the next day, 50 mL of saturated NaCL was added and samples were thoroughly mixed to ensure the fecal pellets were entirely broken up. A Pasteur pipette was used to sample fecal mixture and fill both chambers of a moistened McMaster slide. Slides were allowed to sit for a few minutes and fecal eggs were counted under a microscope. Number of eggs per gram (epg) was calculated by totaling both sides of the McMaster slide and multiplying by 100.

Approximately 12 h after the last loline dose, lambs were slaughtered following stunning with a captive bolt, the abomasum and first 10 m of small intestine was ligated and removed, the number of worms present in abomasum and small intestine were enumerated. Worm burdens were determined separately for *T. circumcincta* in the abomasum and *T. colubriformis* in the small intestine. Following slaughter, the abomasum and small intestine contents were collected, and tissues were washed with water removing any non-adhered worms. The contents and washings were transferred to a beaker; water was added so each had a volume of 2 L. Contents were thoroughly mixed, and four 50 mL subsamples were taken and placed with 20 mL formalin representing 1/10th of the original sample. Washed tissues were then cut up into pieces and digested with 1% pepsin, 1% HCL solution at 38 °C for 16–20 h [26]. After digestion material was passed through a 45 micron sieve, washed with water and fixed in a 5% formalin for storage until worm enumeration. Total digest and wash containers were made up to 100 mL with water and two, 10 mL samples were taken and counted under a microscope. As the wash represented 1/10th of the original sample, the number of worms was determined per mL, multiplied by the containers 100 mL, and multiplied by 10 to yield a final total count. Digests were a total sample, so were only multiplied by the dilution factor (10) to give a total number of worms established in the tissue. Percent establishment was calculated by adding the total number of worms in digest and wash divided by the number of worms dosed to the lambs times 100.

Counts of *T. circumcincta* and *T. colubriformis* were transformed logarithmically to base 10 and analyzed using ANOVA in Genstat (18th edition) between LOL dosed at L4 or adult worm stage and CON-fed lambs.

### 2.5. In Vivo Study 2: Loline Effects against H. contortus

Loline seed extract was used to examine anthelmintic effects of L4 and adult parasite stage of the nematode *H. contortus* in lambs (seven lambs total) approximately 12–16 weeks old (Lincoln University Animal Ethics Committee #2019-07). Lambs were still suckling, and group pens were used to house the lambs with their dams. Ewes and lambs were fed ad libitum lucerne pellets and had free access to water. A creep feeder for the lambs was also set up for ad libitum feed of a lamb muesli. Lambs were treated with an anthelmintic at 2 mL per 15–20 kg (Zolvix Plus, PGG Wrightson, Christchurch, New Zealand) and 10 days later were infected with 20,000 L3 infective *H. contortus*. There were three treatments, loline-treated lambs fed via milk bottle (LOL milk, *n* = 2), loline-treated lambs dosed orally (LOL feed, *n* = 2), and non-loline-treated lambs (CON, *n* = 3).

Similar to in vivo study 1, lambs were trained to drink milk from a bottle with a teat stimulating the esophageal groove reflex. At 10 days post parasite infection, two lambs were offered 100 mL milk twice, 60 h apart containing loline seed extract to supply 52.5 mg/kg LW [14] of loline. The same two lambs were offered loline-laced milk 23 days post larvae infection (LOL milk), and two additional lambs received loline orally dosed with water (LOL feed).

Fecal samples were also taken at the beginning prior to parasite infection, 14 days and every 3 days after until the end of the experiment. Number of eggs per gram (epg) was determined as described in in vivo study 1.

Approximately 12 h after the last loline dose, lambs were slaughtered following stunning with a captive bolt, and the abomasum was ligated and removed, and the number of worms present were counted to determine efficacy using the procedures previously described by in vivo study 1. 

All data were analyzed using Genstat (18th edition). Number of *H. contortus* was transformed logarithmically to base 10 (log10) and analyzed using ANOVA between LOL given through milk or orally dosed and CON-fed lambs. *H. contortus* fecal egg counts were also transformed to log10 and analyzed using REML.

### 2.6. In Vivo Study 3: Oral Dosing Loline and Its Effects against Mixed Infection of L4 T. circumcincta, T. colubriformis, and Adult H. contortus

Following the outcome from the above-mentioned pilot studies, loline seed extract was used to examine anthelmintic effects of L4 stage of *T. circumcincta*, *T. colubriformis* and adult parasite stage of nematode *H. contortus* in weaned lambs (sixteen Coopworth lambs total) approximately 6–8 weeks old (Lincoln University Animal Ethics Committee #2018-34A). Lambs were removed from pasture and housed in individual pens and offered ad libitum lucerne pellets and had free access to water. Feed intake and refusals were recorded daily. Lambs were treated upon housing with an anthelmintic at 2 mL per 15–20 kg (Zolvix Plus, PGG Wrightson, Christchurch, New Zealand) and 10 days later were infected with 20,000 L3 infective *H. contortus* at day 0 and 10,000 *T. circumcincta*, and 10,000 *T. colubriformis* at day 14. Treatments were, loline-treated lambs (LOL, *n* = 7) fed orally, and non-loline-treated lambs (CON, *n* = 7). Additionally, two of the non-loline-treated lambs were fed NIL seed extract orally (NIL, *n* = 2) as a control for the seed extract.

**Figure 1:**
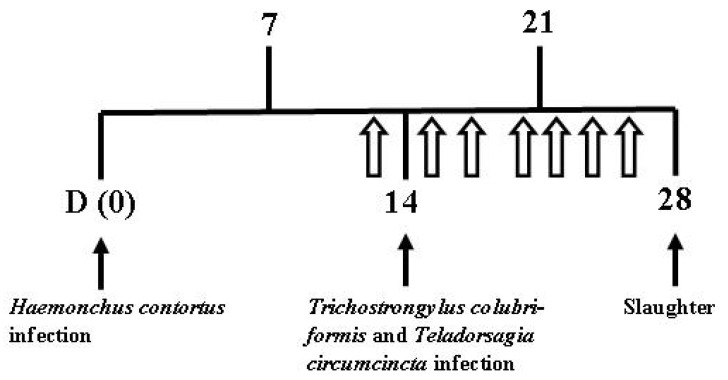
Timeline for lambs (*n* = 16) infected with Haemonchus contortus, Trichostrongylus colubriformis, and Teladorsagia circumcincta and dosed loline/fecal sampled 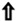.

Loline was delivered at a rate of 52.5 mg/kg LW [14], mixed with water (30 mL) and dosed on days 13, 15, 17, 20, 22, 24, and 26. The day 13 dose was one day prior to *T. circumcincta* and *T. colubriformis* infection (Figure 1). The two lambs receiving NIL seed extract were administered the same amount as lambs receiving LOL treatment.

Fecal samples were taken prior to parasite infection, and at each day of loline doses (Figure 1) and at the day of slaughter. Number of eggs per gram (epg) was determined as described in in vivo study 1.

Approximately 12 h after the last loline dose, lambs were slaughtered following stunning with a captive bolt, the abomasum was removed, and the number of worms present counted to determine efficacy using the procedures previously described by in vitro study 1.

Genstat (18th edition) was used to analyze all data. Worm count data were logarithmically transformed to base 10 for *H. contortus*, *T. colubriformis*, and *T. circumcincta* and analyzed using ANOVA. Fecal egg count data were also log10 transformed and was analyzed using REML. Lamb weight gain and feed intake were analyzed using two-way ANOVA. Significance was declared at *p* ≤ 0.05 and trends at 0.05 ≤ *p* ≤ 0.10.

## 3. Results

### 3.1. In Vitro Experiments

Loline effects on larval migration of *T. colubriformis* in either LOL or NIL extracts are displayed in Table 2. Percent migration was not affected by concentration in either LOL or NIL seed extracts (*p* = 0.29), nor was there an extract x concentration interaction (*p* = 0.52).

Establishment of *T. circumcincta* or *H. contortus* in lambs fed loline is displayed in Table 3. *T. circumcincta* was decreased (*p* < 0.01) by 59% in the abomasal tissues of a lamb fed loline-laced milk (LOL) compared with control (CON). However, establishment of *H. contortus* was unaffected (*p* = 0.49) by loline feeding (Table 3).

### 3.2. In Vivo Studies 1 and 2: Loline Effects against T. circumcincta and T. colubriformis, or H. contortus Establishment

Average worm count of *T. circumcincta*, *T. colubriformis*, or *H. contortus* of CON lambs or lambs treated with loline at L4 or adult worm stage through feed or milk is displayed in Table 4. Percent establishment of *T. circumcincta* was 78%, 75%, and 60% for CON, and lambs treated with loline at adult or L4 worm stage, respectively. However, no statistics were calculated, as there was only one lamb per treatment.

Establishment of *T. colubriformis* was 45%, 80.5%, and 11% for CON, and lambs treated with loline at L4 or adult worm stage, respectively. Lambs treated with loline at L4 were significantly reduced (*p* = 0.02) compared with CON or lambs treated with loline at adult worm stage.

No treatment difference between control and loline dosed in feed or milk was observed for lambs infected with *H. contortus* (*p* = 0.68).

Prior to infection with a mix of *T. circumcincta* and *T. colubriformis*, faecal egg counts were zero. At the end of the experiment, fecal egg counts were similar (*p* = 0.17), with an average of 100, 500, and 225 fecal eggs per gram of feces for lambs treated with loline at L4, or adult worm stage, and CON, respectively.

**Figure 2:**
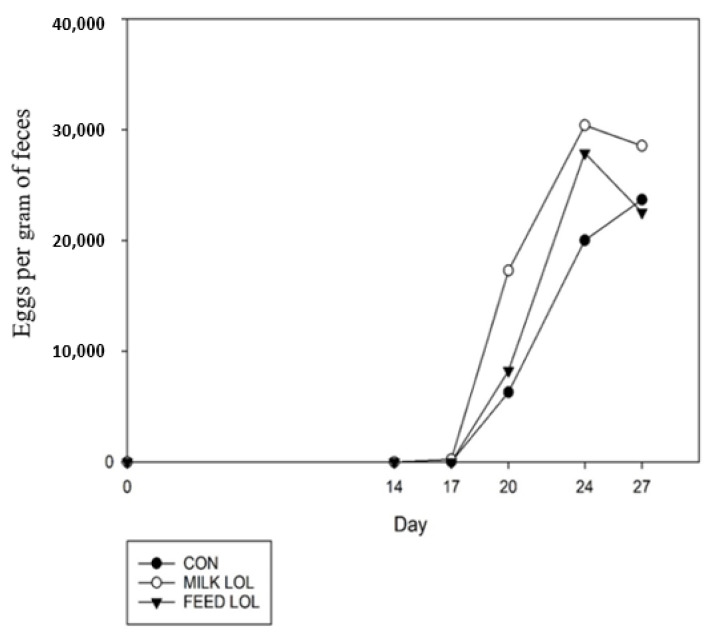
Average fecal egg counts of lambs infected with *Haemonchus contortus* treated with loline either orally or through milk or without.

Fecal egg counts for *H. contortus* infection throughout in vivo trial 2 are displayed in Figure 2. Overall, fecal egg counts increased over time (*p* = 0.01); however, there was no treatment difference (*p* = 0.19) between CON, milk LOL, and feed LOL nor a treatment by day interaction (*p* = 0.82).

### 3.3. In Vivo Study 3: Oral Dosing Loline and Its Effects against Mixed Infection of L4 T. circumcincta, T. colubriformis, and Adult H. contortus

**Figure 3:**
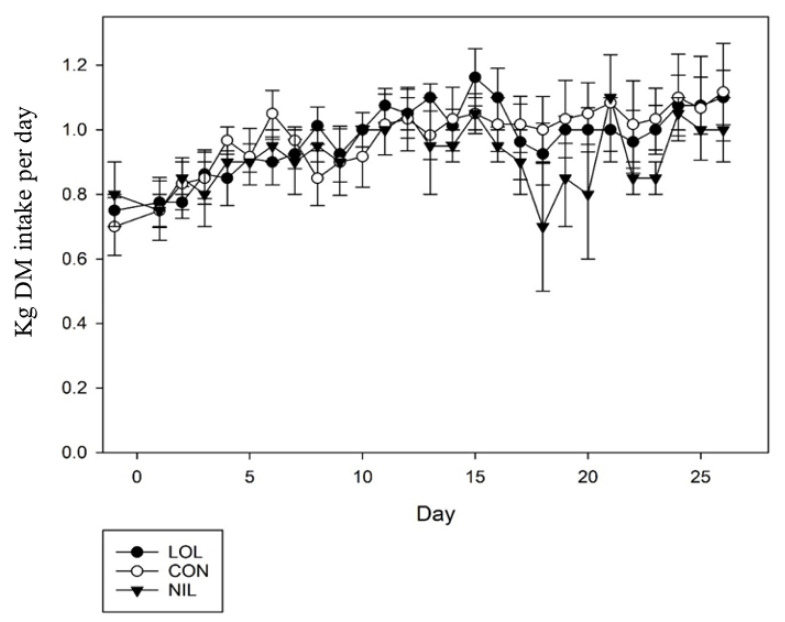
Dry matter intake of lambs with mixed parasite infection treated with a meadow fescue seed extract with (LOL) or without loline (NIL), or non-treated (CON).

Feed intake increased over time (*p* = 0.01, Figure 3) but no treatment differences were observed between LOL, CON and NIL (*p* = 0.18), nor was there a treatment x day interaction (*p* = 0.9).

**Figure 4:**
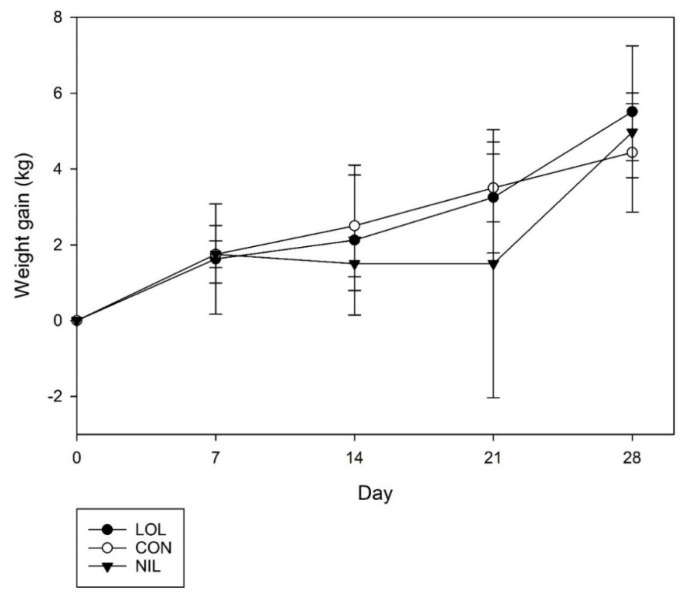
Average weight gain of lambs with mixed parasite infection treated with a meadow fescue seed extract with (LOL) or without loline (NIL), or non-treated (CON).

Average lamb weight gains increased over time (*p* = 0.01, Figure 4) and there were no treatment differences between NIL, CON and LOL (*p* = 0.51) nor was there a treatment by day interaction (*p* = 0.75). Average lamb weight gains were 3.9 ± 0.6, 3.5 ± 0.5, and 2.8 ± 0.6 kg for LOL, NIL and CON, respectively, (*p* = 0.49).

**Figure 5:**
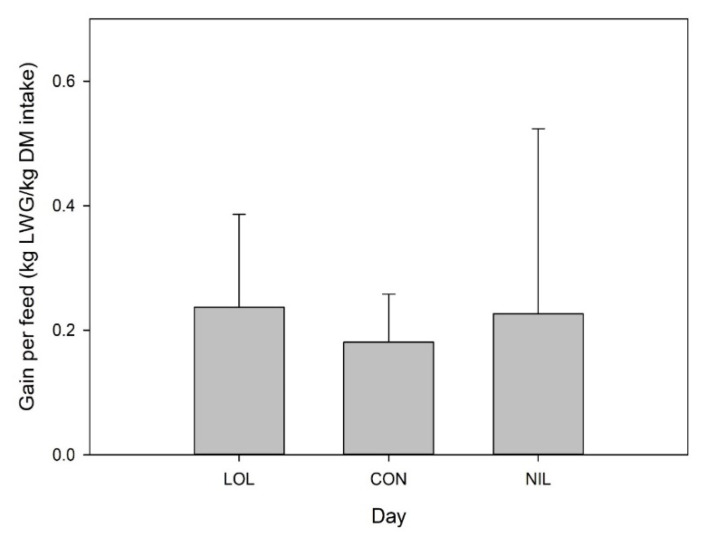
Average growth efficiency over the 28 day trial measured as gain per feed (kg LWG/kg DM intake) of lambs with mixed parasite infection treated with a meadow fescue seed extract with (LOL) or without loline (NIL), or non-treated (CON).

Growth efficiency measured as gain per feed (kg LWG/kg DM intake) varied throughout the trial (*p* = 0.01, Figure 5). Overall, average growth efficiency was 0.18 in CON lambs, which was less (*p* = 0.01) than in LOL- (0.24) or NIL- (0.23)-treated lambs. Overall, day by treatment interaction was significant (*p* = 0.01), with differences detected between days 8–14 and 15–21, where NIL was less than CON or LOL. Between days 22 and 28, treatments varied, with CON greater than LOL or NIL.

**Figure 6:**
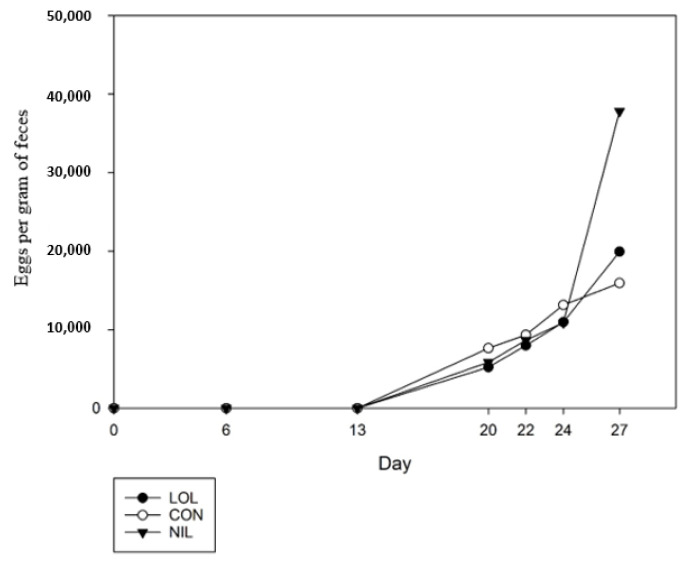
Average fecal egg counts of lambs with mixed parasite infection treated with a meadow fescue seed extract with (LOL) or without loline (NIL), or non-treated (CON).

Fecal egg counts were zero at the start of the trial and increased with time (*p* = 0.01, Figure 6). Overall, there was no treatment difference (*p* = 0.39) between LOL, CON, or NIL nor a treatment by day interaction (*p* = 0.25). Average egg count was 19,925, 15,917, and 37,800 per gram of feces for LOL, CON and NIL, respectively, at slaughter on day 27.

Average worm counts of CON or LOL-treated lambs at L4 stage of *T. circumcincta*, and *T. colubriformis*, or adult *H. contortus* are displayed in Table 5.

Percent establishment of *T. circumcincta* was similar (*p* = 0.96), being 69%, 69.5%, and 69.4% for LOL, CON and NIL, respectively. Similarly, no treatment differences were observed (*p* = 0.43) for *T. colubriformis*-infected lambs, with establishment rates of 24.2%, 27.2%, and 47.8% for LOL, CON, and NIL, respectively. Average worm counts of LOL, CON, or NIL lambs infected with *H. contortus* are displayed in Table 5 and were not significantly different from each other (*p* = 0.15).

## 4. Discussion

Loline had limited anti-parasitic effects, with the only suggestion of an anti-parasitic effect occurring when larvae are in close contact with gastric tissues and mucosal surfaces. Loline had no or minimal effects on in vitro larval migration of L3 *T. colubriformis* and preliminary in vivo results of adult *T. colubriformis* and *T. circumcincta*. For the larval migration assay, this was unexpected as plant extracts containing loline have shown anthelmintic qualities [11,12]. Bacetty et al. [24] found that a combination of NAL and NFL at 100 and 250 µg/mL was nematocidal to the plant nematode *Pratylenchus scribneri*. N-formyl loline and NAL are the principle natural loline alkaloids produced in meadow and tall fescue [13], with NFL being the predominant alkaloid [16]. Combined NFL and NAL composed 86% of the seed extract used in this experiment, and every tested concentration in the larval migration assay would have been greater than 250 µg/mL. Furthermore, egg hatching and larval motility of *T. colubriformis* and *T. circumcincta* are affected by a seed extract of meadow fescue and tall fescue at a high concentration (1:1 ratio of water to seed extract). Meadow fescue and tall fescue both contain loline; however, concentrations were not reported, and results appeared independent of the presence of alkaloid-producing endophyte or endophyte type and identification of causing agent was not explored [12]. These reported concentrations are higher than the offered loline dose in vivo. Lambs were offered a loline dose at 52.5 mg/kg, which, when converted to µg/mL, is the same concentration. It is possible that the dose was too low to have any effect on adult *T. colubriformis* and *T. circumcincta*, although the dose chosen was to reflect the likely consumption in situ when grazing loline pastures [14].

When in close association with blood or tissues, there was some indication of an anthelmintic effect. This was observed in the L4 mucosal browsers *T. colubriformis* and *T. circumcincta* and adult blood feeder *H. contortus* in vivo. Preliminary results showed lambs treated with loline reduced establishment of L4 larvae of *T. circumcincta* and *T. colubriformis* by 75 and 23%, respectively, and adult *H. contortus* by 35% compared with controls. Loline has a documented history of being a feed deterrent in grass grubs and *Pratylenchus scribneri* [11,27,28] and it is possible that the same mechanisms may be at play here. Furthermore, it is suggested that the mode of action of alkaloids is antagonizing receptors in the central nervous system, causing death [29,30,31]. However, loline has no documented affinity to a variety of central nervous system receptors [32,33,34] and is unlikely to cause parasite death. Main routes of administration of any chemical to have an effect against a parasite are oral ingestion or diffusion through its external surfaces, which is dependent on molecule lipophilicity [35,36]. Based on the characteristics (hydrophilicity, neutral charge, and low molecular weight) of loline, diffusion through a parasite’s external surfaces would be limiting, but it would be an ideal molecule for clearance to underlying mucous gel layers in the gastrointestinal tract [37]. Loline is also found in the blood of horses and sheep dosed orally [15,38], meaning loline has good location availability (blood and gastric mucous layers) to be a possible feed deterrent. Although concentrations in mucous were not measured, this could explain the decreased mucosal browsers *T. colubriformis* and *T. circumcincta* and adult blood feeder *H. contortus* in vivo as well as the reduced in vitro establishment of *T. circumcincta* more than 12 h after the last loline dose in lamb tissues.

Unexpectedly, preliminary results (in vivo study 1 and 2) contrasted with further investigations (in vivo study 3). This potentially reflects mode of ingestion or the length of time loline had contact with L4 mucosal browser or adult *H. contortus*. Orally dosed loline had minimal effects on worm counts and fecal egg counts of a mix of L4 *T. colubriformis*, *T. circumcincta* and adult *H. contortus*. Concentration and target availability influence maximum drug efficacy on parasites [35,36]. In the preliminary trials, loline was dosed in the abomasum through milk and esophageal groove closure. Based on Froehlich [15], loline appears to be minimally destroyed and/or absorbed out of the rumen and it was decided that dosing via abomasum was not necessary. However, oral dosing could have resulted in loline becoming diluted or associated with rumen contents, reducing available concentration for parasites in the abomasum and small intestine. It is known that short fasting or temporary reduced feeding reduces gastric transit time, increasing plasma availability and anthelmintic efficacy in calves and sheep [39,40,41]. If loline became associated with rumen contents, this could have sped up its excretion or made less available for absorption into the gastric mucous layer or blood, influencing its effect on parasites. In addition, slaughtering animals sooner could have shortened loline’s availability in mucus surfaces and thus contact time with the parasites. In the preliminary in vivo study 1, *T. colubriformis* and *T. circumcincta* larvae were treated with loline at L4 and were slaughtered 16 days later, whereas lambs in in vivo study 3 were slaughtered 3 days after developing to L4. Loline is excreted in urine of sheep for up to 60 h after dosing [14] and it is possible that longer contact is needed to provide an anthelmintic and detect an effect on *T. colubriformis* and *T. circumcincta*.

The effects observed, when they were observed, can be attributed to loline. In vivo study 3 using a seed extract with (LOL) and without (NIL) loline to potentially identify and isolate loline effects on parasites showed the NIL extract to have little effect on the average worm counts of *T. circumcincta*, *T. colubriformis*, or *H. contortus*, or the fecal egg count. Plants naturally contain a variety of compounds [42] and a disadvantage of crude plant extracts is purification and isolating the effects of the compounds. Loline is no different as its isolation from plants and creation of synthetic loline is difficult [43]. From the study, NIL extract had little effect on parasites, suggesting that any observed anti-parasitic effect was due to loline. However, many secondary metabolites and plant compounds have anti-parasitic effects [1] and can work in synergy with each other [6]. In one case, investigating effects of quinolizidine and piperidine alkaloids on *H. contortus* and *T. circumcincta*, some of the non-alkaloid seed extracts were better, suggesting that non-alkaloid components have an effect [30]. As plant breeding can change primary and secondary metabolites in the plant [27,44,45], future research may need to isolate the effects of loline in meadow fescue.

The form of loline may be important. Based on Froehlich [15], loline metabolites are generally unconverted in ruminal fluid; but of the forms of loline, only loline base and NFL were passively transported across all gastrointestinal epithelial [15]. Furthermore, although all five loline metabolites are excreted in lamb urine, excretion peaks at 2 h post dosing, after which the predominant metabolites are loline base and NFL [14]. Similarly, only NFL and loline base have been found in the blood plasma of lambs [15] and some NFL and NAL in horse serum [38]. This may be expected as NFL is the predominant metabolite found in meadow fescue and tall fescue [16,46] and has been noted as a strong insecticide to several orders of insects [17,47]. However, some research has pointed to NAL [27] or a combination of NAL and NFL [24] to have some insecticidal and plant parasitic effects, respectively. It is possible that other forms of loline may be effective but have not been reported/studied due to low natural concentrations. Based on the thesis of Froehlich [15], it could be proposed that NFL is likely the most effective form, assuming simple loline base is an inactive loline form which metabolites degrade to. N-formyl loline is the metabolite of greatest concentration in seed extract [15] and would have the greatest bioavailability to affect parasites as it is slowly excreted in urine [14] and is found in blood for many hours after dosing [15] compared with other loline metabolites.

## 5. Conclusions

In conclusion, there was limited evidence to support an anti-parasitic effect of loline, but it remains possible that loline may have some deterrence to parasites that are in close contact with the gastric mucous layers or blood. Preliminary in vivo results showed that loline reduces the establishment of L4 mucosal *T. colubriformis* and *T. circumcincta*, and adult blood feeder *H. contortus* when dosed via milk. However, this contrasted to when loline was orally dosed, possibly a reflection of the concentration of loline being diluted by rumen contents and decreased target availability, an effect which may have reflected the early ending of the trial, resulting in lower deterrence.

## Figures and Tables

**Table 1:** Loline alkaloid effects on gastrointestinal nematodes trial outline.

In Vitro Studies	Parasite(s) Studied
Study 1: Larval migration	L3 *T. colubriformis*
Study 2: Abomasal establishment	L3 *T. circumcincta*
Study 3: Abomasal establishment	L3 *H. contortus*
**In Vivo Lamb Establishment Studies**	**Parasite(s) Studied**
Study 1 (*n* = 8)	L4 and adult *T. circumcincta* and *T. colubriformis*
Study 2 (*n* = 7)	L4 and adult *H. contortus*
Study 3 (*n* = 16)	L4 *T. circumcincta* and *T. colubriformis,* and adult *H. contortus*

**Table 2:** In vitro effects of different concentrations of loline on larval migration (LM) of *Trichostrongylus colubriformis*.

	Concentration, ppm			
Sample	16,000	8000	4000	2000
LOL, % LM	87.2 ± 9.0	85.5 ± 6.1	88.8 ± 6.8	91.2 ± 5.8
NIL, % LM	59.7 ± 20.6	85.0 ± 3.5	81.2	93.2

**Table 3:** In vitro percent establishment of *Teladorsagia circumcincta* (*n* = 2) or *Haemonchus contortus* (*n* = 3) in lambs treated with or without loline.

	Average % Establishment	
	LOL	CON
*Teladorsagia circumcincta*	30.8 ± 2.4 ^a^	74.8 ± 2.3 ^b^
*Haemonchus contortus*	56.5 ± 4.4	50.5 ± 8.3

^a,b^ Means within rows with unlike superscripts differ, *p* < 0.05.

**Table 4:** Average worm count of *Trichostrongylus colubriformis*, *Teladorsagia circumcincta*, or *Haemonchus contortus* of CON lambs or lambs treated with loline at L4, or adult worm stage, through feed or milk.

	Average Worm Count			
	LOL, L4 Dose	LOL, Adult Dose		CON
		Milk Fed	Feed Fed	
^1^ *Teladorsagia circumcincta*	2985	3765		3890
^1^ *Trichostrongylus colubriformis*	1137 (589–1687) ^a^	8050 (7810–8290) ^b^		4473 (2933–4180) ^b^
^2^ *Haemonchus contortus*		18,750 (16,875–20,625)	19,250 (12,750–25,750)	29,125 (16,875–49,875)

^a,b^ Means within rows with unlike superscripts differ, *p* < 0.05. ^1^ In vivo study 1 (*n* = 8): loline effects against *T. circumcincta* and *T. colubriformis*. ^2^ In vivo study 2 (*n* = 7): loline effects against *H. contortus.*

**Table 5:** Average worm counts of lambs treated with a meadow fescue seed extract with (LOL, *n* = 7) or without loline (NIL, *n* = 2), or non-treated (CON, *n* = 7) at L4 or adult worm stage of *Teladorsagia circumcincta*, and *Trichostrongylus colubriformis*, or adult *Haemonchus contortus*.

	Average Worm Counts		
	LOL	CON	NIL
*Teladorsagia circumcincta*	6900 (2700–10,000)	6951.7 (2960–9810)	6940 (6280–7600)
*Trichostrongylus colubriformis*	2415 (100–4450)	2720 (490–5590)	4775 (3190–5590)
*Haemonchus contortus*	14,630 (6650–29,090)	7938 (440–11,590)	29,750 (23,810–35,690)

## Data Availability

Data is included within the article.
